# The association between glycemia and clinical outcomes in patients with diabetes mellitus and pulmonary thromboembolism

**DOI:** 10.20945/2359-3997000000544

**Published:** 2023-01-17

**Authors:** Gülru Polat, Mutlu Onur Güçsav, Özer Özdemir, Merve Ayik Türk, Damla Serçe Unat, Dursun Tatar

**Affiliations:** 1 Dr. Suat Seren Chest Diseases and Surgery Training and Research Hospital Department of Pulmonology Izmir Turkey Department of Pulmonology, Dr. Suat Seren Chest Diseases and Surgery Training and Research Hospital, Izmir, Turkey; 2 Izmir Tepecik Training and Research Hospital Department of Pulmonology Izmir Turkey Department of Pulmonology, Izmir Tepecik Training and Research Hospital, Izmir, Turkey; 3 Izmir Bozyaka Training and Research Hospital Department of Pulmonology Izmir Turkey Department of Pulmonology, Izmir Bozyaka Training and Research Hospital, Izmir, Turkey; 4 Kemalpaşa Hospital Department of Pulmonology Izmir Turkey Department of Pulmonology, Kemalpaşa Hospital, Izmir, Turkey; 5 Health Science University Dr. Suat Seren Chest Diseases and Surgery Training and Research Hospital Department of Pulmonology Izmir Turkey Department of Pulmonology, Health Science University Dr. Suat Seren Chest Diseases and Surgery Training and Research Hospital, Izmir, Turkey

**Keywords:** Diabetes mellitus, mortality, recurrence, pulmonary thromboembolism, clinical class of embolism

## Abstract

**Objective:**

Various studies have shown that diabetes mellitus (DM) increases the risk of thrombosis in the venous system as well as in the arterial system. In this study, it was aimed to evaluate the association between admission blood glucose levels and clinical severity, recurrence, and mortality in pulmonary embolism in patients with DM.

**Materials and methods:**

This study was designed as a retrospective cross-sectional study. Patients with DM who were admitted to a tertiary care hospital due to pulmonary embolism (PE) between 2014 and 2019 were included. Demographic characteristics, radiological findings, clinical class of embolism, and mortality data were retrieved from hospital records. Patients with and without recurrent disease, as well as patients who survived and died, were compared. Also, patients were classified according to quartiles of admission blood glucose levels. The quartiles were compared in terms of mortality, clinical, class, and recurrence.

**Results:**

Two hundred ninety-three patients with DM and PE were included in the study. Patients with adverse outcome had significantly higher admission blood glucose levels (respectively, 197.9 ± 96.30 mg/dL vs. 170.7 ± 74.26 mg/dL; p = 0.03). Patients in the third and fourth quartile of admission blood glucose levels (>152 mg/dL) had significantly more severe disease with a higher proportion of massive and sub-massive PE and higher pro-BNP levels (respectively, p = 0.01 and 0.02).

**Conclusion:**

Non-survived patients and recurrent disease were associated with higher admission blood glucose levels. Also, patients with admission blood glucose levels higher than 152 mg/dL tend to have clinically more severe diseases.

## INTRODUCTION

Diabetes mellitus (DM) represents a major cause of increased cardiovascular disease risk (1). Hyperglycemia is found to be associated with arterial thrombosis (2,3). Although there are still controversial data, several studies showed that this effect is also relevant for venous thromboembolism (VTE) (4). The three components of the Virchow triad, stasis, endothelial injury, and hypercoagulability are found to be associated with hyperglycemia, thereby increasing the risk of VTE in patients with DM (5,6). Endothelial injury leads to the activation of coagulation factors such as the platelets, prothrombin, and thrombin-antithrombin complex. Also, it causes partial inhibition of the fibrinolytic system, owing to the subsequent formation of resistant clots as well as increased levels of plasminogen activator inhibitor-1 (PAI-1) (7,8). Coagulation factors, like fibrinogen, von Willebrand factor, and D-dimer are elevated in patients with DM (9). Both acute and chronic hyperglycemia may contribute to this process (10).

However, the risk of VTE associated with DM is thought to be significantly lower compared to other risk factors for VTE such as surgery, fractures in the lower extremities, spinal cord injury, cancer, and chemotherapy (11). Furthermore, some reports did not find any significant association between DM and VTE (12,13).

Eighty percent of patients with type-2 DM die due to thrombotic events. Approximately 75% of these deaths result from acute myocardial infarction (MI), while the remaining are caused by different peripheral vascular disorders, mainly cerebrovascular diseases (14). Early and tight blood glucose control has been shown to lower cardiovascular disease risk and mortality in DM patients (10). Hyperglycemia is a major indicator of poor prognosis in patients with cardiovascular disorders (15). Despite numerous studies examining the effect of hyperglycemia on cardiovascular disease risk and mortality, studies on the association between hyperglycemia and pulmonary embolism (PE) are relatively scarce, with only a limited number of published reports evaluating the association of DM and/or hyperglycemia with recurrence, clinical classification (i.e., massive, sub-massive, and non-massive), and mortality of PE (8,16).

The primary aim of this study was to evaluate the association of admission blood glucose levels with clinical severity, recurrence, and mortality of pulmonary embolism in patients with DM.

## MATERIALS AND METHODS

This study was designed as a retrospective cross-sectional study. DM patients diagnosed with PE in a tertiary care unit between 1^st^ July 2014 and 1^st^ July 2019 were included in the study. In all patients, the diagnosis of pulmonary embolism was confirmed by filling defects of pulmonary arteries in computed tomography pulmonary angiography (CTPA), or mismatched defects seen in ventilation and perfusion pulmonary scintigraphy. Additional diagnostic information was obtained from clinical, echocardiographic (ECHO), and radiological evaluations, as well as D-dimer measurements (above the normal limit, 0.5 μg/mL) and blood gas analysis. Demographic data, Charlson comorbidity index, blood glucose levels at presentation, routine blood chemistry, radiological findings, diagnostic modalities, ECHO findings, co-existing deep venous thrombosis (DVT), disease severity (massive, sub-massive, non-massive), mortality, and recurrence data were recorded. The disease classification was based on vital, clinical, and ECHO findings, as described in the European Cardiology Society (ESC) and European Respiratory Society (ERS) conjoint Guidelines for the Diagnosis and Treatment of Pulmonary Embolism (11). The clinical nature of the pulmonary embolism was categorized according to the presence or absence of known risk factors associated with thromboembolism, as provoked and unprovoked PE (i.e., whether the event was provoked by a known risk factor or not) (17).

Exclusion criteria were age under 18 years and embolism due to non-thrombotic causes (amniotic fluid embolism, tumor embolism, etc.). Also, cases with incomplete and missing data were excluded.

Data were analyzed using Statistical Package for the Social Sciences (SPSS) Version 22 software program (SPSS Inc., Chicago, IL, USA). A priori statistical power analysis was conducted to test the difference of dichotomous variables in two independent groups using a two-tailed test, a medium effect size (δ = 0.50), and an alpha of 0.05 with a power of 0.8. Results showed a total sample of 240 patients. Demographic, clinical, imaging, and laboratory findings were expressed using descriptive statistics. The results were presented as number (n), percent (%), and mean ± standard deviation (SD). Patients who had recurrent diseases in the follow-up period or died during their management period were classified to have an adverse outcome. Independent samples t-test, X^2^ test, or Mann Whitney-U test were used to compare patients with and without adverse outcomes, according to the properties and distribution of variables.

Patients were also classified as quartiles of admission blood glucose levels. Chi-square analyses were used for the comparison of categorical variables between independent groups. The normal distribution of numerical variables was tested with the Kolmogorov-Smirnov test. Variables with normal distribution were compared using one-way analysis of variance (ANOVA) analysis, while Kruskal-Wallis analysis was used for those without normal distribution. p level of < 0.05 was considered statistically significant.

## RESULTS

**Figure 1:**
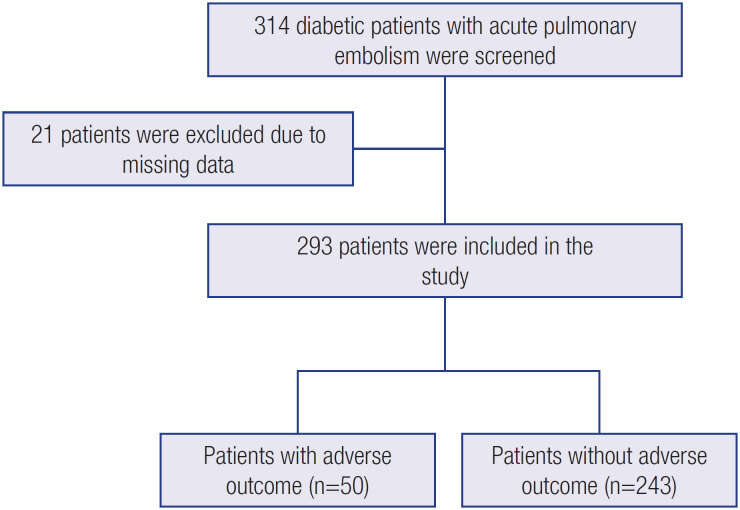
Flowchart diagram for the study population. Adverse outcomes were death or recurrence of pulmonary embolism.

Three hundred and fourteen patients with the diagnosis of pulmonary embolism and DM were screened for the study. Twenty-one patients were excluded due to missing data. The study population was composed of the remaining 293 patients. The flowchart of the study population is presented in Figure 1.

The mean blood glucose level at presentation was 175.4 ± 78.94 mg/dL. The mean age of the study population was 67.0 ± 12.0 years. Table 1 summarizes the demographic and clinical characteristics of the patients. Accordingly, 59.4% of the patients were female. The most common symptom was shortness of breath (79.5%), followed by chest pain (24.9%), and cough (22.9%). The most frequently used diagnostic modality was thoracic CT angiography (74.7%). PE was mostly located in segmental branches of pulmonary arteries (27.5%). Postero-anterior chest X-rays used as an adjunctive diagnostic modality did not show any signs suggestive of PE in 47.4% of the patients, while cardiomegaly (40%) and pleural effusion (33%) were the most common chest x-ray manifestations. The mean D-dimer in PE patients was 3704.4 ± 3489.43 ng/mL, and the mean platelet count was 282.544.67 ± 112.209.19. The mean of the Charlson comorbidity index for the study population was 4.8 ± 2.1. Overall, 69.6% of the patients had least at least one risk factor other than DM. Concomitant DVT was present in 77 patients (26.3%). The most common site of DVT was the popliteal vein (n = 51, 66.2%). Forty-four patients (15%) had a recurrence of pulmonary embolism. Of the 25 patients (8.5%) with massive PE, 18 (6.1%) had received thrombolytic therapy, and 8 (2.7%) died due to PE during the clinical course. All patients receiving thrombolytic therapy had recombinant tissue plasminogen activator (rtPA) as the thrombolytic agent.

**Table 1:** Demographic and clinical characteristics of the study population

Characteristic		n = 293
Age, mean ± SD		67.0 ± 12.0
Gender		
	Female, n (%)	174 (59.4)
	Male, n (%)	119 (40.6)
Cigarette smoking		
	Non-smoker, n (%)	70 (23.9)
	Ex-smoker, n (%)	44 (15.0)
	Smoker, n (%)	41 (14.0)
Non-DM comorbidity		
	Hypertension, n (%)	176 (60.1)
	Cardiovascular disease, n (%)	85 (29.0)
	Heart failure, n (%)	42 (14.3)
	Cancer, n (%)	42 (14.3)
	COPD, n (%)	50 (17.1)
	Morbid obesity, n (%)	21 (7.2)
	Cerebrovascular disease, n (%)	7 (2.4)
	Other, n (%)	64 (21.8)
Charlson Comorbidity Index, mean ± SD		4.8 ± 2.1
Presence of precipitating risk factors for thromboembolism		
	Provoked, n (%)	204 (69.6)
	Unprovoked, n (%)	89 (30.4)
Symptoms		
	Shortness of breath, n (%)	233 (79.5)
	Chest pain, n (%)	73 (24.9)
	Cough, n (%)	67 (22.9)
	Back pain, n (%)	34 (11.6)
	Calf swelling, n (%)	17 (5.8)
	Calf pain, n (%)	14 (4.8)
	Hemoptysis, n (%)	12 (4.1)
	Syncope, n (%)	12 (4.1)
	Other, n (%)	41 (14.0)
Risk factors		
	Immobilization, n (%)	55 (18.8)
	Cancer, n (%)	42 (14.3)
	Surgery in the past 1 month, n (%)	38 (12.1)
	Obesity, n (%)	21 (7.2)
	Hereditary thrombophilia, n (%)	16 (5.5)
	Trauma, n (%)	3 (1)
	Oral contraceptive usage, n (%)	1 (0.3)
	Pregnancy, n (%)	1 (0.3)
	Other, n (%)	2 (0.6)
	Deep vein thrombosis, n (%)	77 (26.3)
Clinical severity		
	Massive embolism, n (%)	25 (8.5)
	Sub-massive embolism, n (%)	68 (23.2)
	Non-massive embolism, n (%)	181 (61.8)
Localization of thrombus		
	Saddle embolism, n (%)	7 (2.4)
	Main pulmonary arteries, n (%)	78 (26.6)
	Lobar pulmonary arteries, n (%)	60 (20.5)
	Segmental – subsegmental pulmonary arteries, n (%)	146 (49.8)
Recurrence, n (%)		44 (15)
Exitus, n (%)		8 (2.7)

SD: standard deviation; DM: diabetes mellitus; COPD: chronic obstructive pulmonary disease.

Fifty patients (17.1%) had adverse outcomes, either recurrent disease or death. Patients with adverse outcomes had significantly higher admission blood glucose levels, compared with patients without adverse outcomes (respectively, 197.9 ± 96.30 mg/dL *vs*. 170.7 ± 74.26 mg/dL; p = 0.03). Demographic, clinical, and laboratory parameters across both groups were similar. The comparison of patients with and without adverse outcomes is presented in Table 2.

**Table 2:** Comparison of study population according to the presence of adverse outcomes (recurrence or mortality)

	Patients without adverse outcomes (n = 243)	Patients with adverse outcomes (n = 50)	p
Age, mean ± SD	67.2 ± 11.90	66.3 ± 12.57	0.65
Gender, female, n (%)	145 (59.7)	29 (58)	0.88
Charlson comorbidity index, mean ± SD	4.9 ± 2.15	4.6 ± 2.01	0.41
Concomitant DVT, n (%)	65 (29.3)	12 (26.7)	0.86
Unprovoked PE, n (%)	74 (30.5)	15 (30)	1.00
Thrombus in main pulmonary arteries, n (%)	70 (29)	15 (30)	0.87
Blood glucose, mg/dL, mean ± SD	170.7 ± 74.26	197.9 ± 96.30	**0.03**
Leucocytes, /mm^3^	9900 (4800)	10400 (3200)	0.61
Hemoglobin, g/dL, mean ± SD	11.9 ± 2.08	12.2 ± 1.85	0.38
Platelets, /mm^3^	261000 (132000)	259000 (138000)	0.55
CRP, mg/dL	2.9 (7.4)	2.1 (3.0)	0.40
D-dimer, ng/mL	2388 (3680)	3019 (5484)	0.08
Pro-BNP, pg/mL	752.1 (3915.5)	1511 (4852.5)	0.17

Data are presented as median (interquartile range) if otherwise is not stated.

SD: standard deviation; PE: pulmonary embolism; DVT: deep vein thrombosis; CRP: C- reactive protein; BNP: brain natriuretic peptide.

Comparison quartiles according to the admission blood glucose levels of patients revealed no differences regarding recurrent disease or survival (respectively, p = 0.20 and p = 0.39). The patients in the third and fourth quartiles (blood glucose levels 153-207 mg/dL and > 207 mg/dL) had significantly more severe disease compared with the patients with lower blood glucose levels (p = 0.01). Charlson comorbidity scores across quartiles were similar but patients in the third quartile were significantly older than other patients (respectively, p = 0.29 and p= 0.02). The comparison of quartiles of admission blood glucose levels is presented in Table 3. Although patients in the third and fourth quartile had more main pulmonary artery thromboembolism, this difference did not reach a statistical significance level (p = 0.18). Also, the comparison of the location of the thrombus did not reveal any significant difference in admission blood glucose levels (Table 4).

**Table 3:** Comparison of clinical and laboratory parameters according to the quartiles of blood glucose levels of the study population at presentation

	Quartile 1 (<122 mg/dL)	Quartile 2 (122-152 mg/dL)	Quartile 3 (153-207 mg/dL)	Quartile 4 (>207 mg/dL)	p
Age, mean ± SD	64.2 ± 13.05	67.3 ± 10.32	70.2 ± 12.06	66.4 ± 11.88	**0.02**
Gender, female, n (%)	39 (54.2)	39 (53.4)	51 (68.9)	45 (60.8)	0.19
Charlson comorbidity index	5.0 (3)	5.0 (2)	5.0 (3)	5.0 (3)	0.29
**Clinical severity**					
Massive or sub-massive PE, n (%)	21 (30.9)	13 (19.7)	28 (40.0)	31 (44.3)	0.01
Concomitant DVT, n (%)	17 (24.3)	17 (25.8)	27 (39.7)	16 (25.4)	0.15
Thrombus location					
Main pulmonary arteries, n (%)	17 (24.3)	19 (26.0)	29 (39.2)	20 (27.0)	0.18
**Laboratory parameters**					
Leucocytes, /mm^3^	9350 (3325)	9850 (6050)	10200 (4850)	10700 (4600)	0.11
Hemoglobin, gr/dL, mean ± SD	12.4 ± 1.84	11.8 ± 1.80	11.7 ± 2.04	11.7 ± 2.39	0.12
Platelets, /mm^3^	276000 (124250)	275500 (154750)	243000 (132000)	256000 (107500)	0.52
CRP, mg/dL	1.7 (3.6)	3.2 (7.9)	2.3 (5.0)	4.5 (8.4)	0.05
D-dimer, ng/mL	2370 (3107)	2531 (3283)	2881 (5738)	2500 (3694)	0.39
Pro-BNP, pg/mL	415.5 (1283.6)	513 (1964.7)	1112.5 (4821.5)	2393 (6070.5)	0.02
Recurrence, n (%)	9 (12.5)	7 (9.6)	12 (16.2)	16 (21.6)	0.20
Mortality, n (%)	2 (2.8)	1 (1.4)	4 (5.4)	1 (1.4)	0.39

Data are presented as median (Interquartile range) if otherwise is not stated.

PE: pulmonary embolism; DVT: deep vein thrombosis; SD: standard deviation; CRP: C- reactive protein; BNP: brain natriuretic peptide.

**Table 4:** Comparison of admission blood glucose levels and location of pulmonary embolism

Location of pulmonary embolism	Admission blood glucose level (mg/dL)	p-value
Main pulmonary arteries (n = 85)	179.7 ± 74.18	0.22
Lober pulmonary arteries (n = 60)	191.1 ± 94.16	
Segmental pulmonary arteries (n = 85)	171.2 ± 74.05	
Sub-segmental pulmonary arteries (n = 61)	162.8 ± 74.03	

In patients in the third quartile of admission blood, glucose levels were associated with older patients compared with other groups (p = 0.02). Charlson comorbidity index and distribution of gender were similar across groups (respectively, p = 0.29 and p = 0.19). In patients in the fourth quartile of admission blood, glucose levels were found to be associated with higher C-reactive protein (CRP) levels at the level of significance and significantly higher pro-brain natriuretic peptide (pro-BNP) levels (p = 0.05 and p = 0.02). Other laboratory parameters were similar across quartiles (Table 3).

## DISCUSSION

In this study, DM patients with acute pulmonary embolism had a mean blood glucose level of 175.4 ± 78.94 mg/dL at admission. Admission blood glucose levels of non-survived patients and patients with recurrent disease were found to be significantly higher. Also, blood glucose levels above 152 mg/dL were associated with more severe disease, with significantly higher pro-BNP levels. It should be kept in mind that the age distribution of the blood glucose level quartiles was not uniform, as patients with admission blood glucose levels between 152-207 mg/dL were significantly older, and this may have some effect on results.

These findings support that hyperglycemia is associated with more severe disease, similarly to previous reports and this association may also be relevant for recurrent disease (16,18). In the study of Zhang and cols., the risk of recurrence among hyperglycemic DM patients was 1.38-fold and 1.34-fold higher as compared to non-hyperglycemic DM patients (95% CI: 1.12-1.55, p = 0.01) and non-diabetic patients (95% CI: 1.09-1.63, p = 0.025) (16). The main findings of the study indicate that although DM alone was not a significant factor for recurrence, DM-associated hyperglycemia increased the risk for recurrence. The main mechanism of this association is not clear. An in vitro analysis of platelets showed that hyperglycemia increases glucose utilization by platelets, leading to increased platelet activation (19). Also, hyperglycemia is thought to alter the coagulation cascade and fibrinolytic system, via increased inflammatory markers as well as through the effect of mediators such as PAI-1 and tissue factor (TF) (20). In hyperglycemic patients, it has been shown by Galanaud and Kahn that certain polymorphisms in genes encoding PAI-1 and platelet endothelium cellular adhesion molecule-1 (PECAM-1) decrease the lysis of thrombus and increase the post-thrombotic events such as acute/recurrent PE following DVT (21).

Elevated mean admission blood glucose levels of the study population were in accordance with previous reports of hyperglycemia in patients with acute pulmonary embolism (22). Altabas and cols. also reported that hyperglycemia was also associated with localization of the thrombus, as bigger thrombus sizes were located more proximally in the pulmonary arterial system (23). Increased inflammation may increase the thrombotic burden and may lead to the formation of larger emboli in diabetic patients. These more voluminous emboli may obstruct more proximal branches of the pulmonary arteries. Although clinically severe disease status was associated with hyperglycemia and thrombus in main and lobar pulmonary arteries tending to have higher blood glucose levels in this study, we found no significant association between embolus localization and hyperglycemia.

Until now, no studies have directly examined the clinical PE class and blood glucose levels at presentation. In Altabas and cols.'s study, hyperglycemia was found to be associated with PESI (Pulmonary Embolism Severity Index) scores, while an association with the mean pulmonary artery pressure (PAP) was reported by Gohbara and cols. (23,24). Only limited literature data indicate a potential relationship between blood glucose at presentation and PE mortality (18,22). On the other hand, in de Miguel-Díez and cols.'s study, the presence of DM did not emerge as an independent risk factor for mortality in PE (25). Akirov and cols. observed a link between hyperglycemia and mortality in non-diabetic patients, but this association was not present in DM patients (26). Our results indicate that there is an association between higher admission glucose levels and adverse outcomes in PE patients. Higher rates of mortality during acute PE in diabetic patients have been accounted for by the increased occurrence of comorbid conditions in these patients, mainly including cardiovascular disorders, cancer, and anemia. Such comorbidities may aggravate the clinical course with a subsequent increase in mortality. We did not find any difference in the Charlson comorbidity index between study groups. However, further well-designed studies with appropriately selected patients are warranted to definitively establish whether hyperglycemia represents an independent risk factor for mortality and recurrence in acute PE.

Previously, an association between higher glucose levels at presentation and increased DVT risk has been demonstrated (4). In this study, there was a tendency for higher concomitant DVT in patients with admission blood glucose levels between 153-207 mg/dL. But this association was not significant.

CRP is an acute phase protein and is a major inflammatory marker. DM patients have been shown to have elevated serum CRP levels, and increased CRP levels have been shown to be a risk factor for DM development in those without DM (27). In our patient population patients with higher blood glucose levels tend to have higher CRP levels at the level of significance. Also, higher pro-BNP levels are in accordance with more severe disease that causes cardiovascular overload.

Our study had certain limitations. Firstly, it was designed retrospectively. Hemoglobin A1c (HbA1c) levels could not be retrieved from patient files, implying that no information could be gathered regarding the blood glucose profiles within the past 2-3 months period. Also, the treatments used for diabetes control and duration of diabetes were not available.

In conclusion, elevated admission blood glucose levels may be associated with more severe disease in diabetic patients with acute PE. Also, this association may be relevant for recurrent and mortal diseases. Further prospective studies are needed to determine the risk of hyperglycemia for recurrent and mortal disease in acute PE.
